# Effect of direct-acting antivirals on the titers of human pegivirus 1 during treatment of chronic hepatitis C patients

**DOI:** 10.1128/spectrum.00641-24

**Published:** 2024-07-25

**Authors:** Ulrik Fahnøe, Lone Wulff Madsen, Peer Brehm Christensen, Christina Søhoel Sølund, Sarah Mollerup, Mette Pinholt, Nina Weis, Anne Øvrehus, Jens Bukh

**Affiliations:** 1 Copenhagen Hepatitis C Program (CO-HEP), Department of Infectious Diseases, Copenhagen University Hospital, Hvidovre, Denmark; 2 Copenhagen Hepatitis C Program (CO-HEP), Department of Immunology and Microbiology, Faculty of Health and Medical Sciences, University of Copenhagen, Copenhagen, Denmark; 3 Department of Infectious Diseases, Odense University Hospital, Odense, Denmark; 4 Institute for Regional Health Research, University of Southern Denmark, Research Unit for Internal Medicine Kolding Hospital, Kolding, Denmark; 5 Clinical Institute, University of Southern Denmark, Odense, Denmark; 6 Department of Infectious Diseases, Copenhagen University Hospital, Hvidovre, Denmark; 7 Department of Clinical Microbiology, Copenhagen University Hospital - Amager and Hvidovre, Hvidovre, Denmark; 8 Department of Clinical Medicine, Faculty of Health and Medical Sciences, University of Copenhagen, Hvidovre, Denmark; Indian Institute of Science, Bangalore, Karnataka, India

**Keywords:** human pegivirus, HPgV-1, hepatitis C virus, HCV, coinfection, direct-acting antivirals, DAA, sofosbuvir, metagenomics, pegylated interferon

## Abstract

Coinfections with human pegivirus 1 (HPgV-1) are common in chronic hepatitis C virus (HCV) patients. However, little is known about whether HPgV-1 is affected by direct-acting antivirals during HCV treatment. Metagenomic analysis and reverse transcriptase-quantitative PCR (RT-qPCR) were performed on RNA from the plasma of 88 selected chronic HCV patients undergoing medical treatment. Twenty (23%) of these HCV patients had HPgV-1 coinfections and were followed by RT-qPCR during treatment and follow-up to investigate HPgV-1 RNA titers. Recovered sequences could be assembled to complete HPgV-1 genomes, and most formed a genotype 2 subclade. All HPgV-1 viral genomic regions were under negative purifying selection. Glecaprevir/pibrentasvir treatment in five patients did not consistently lower the genome titers of HPgV-1. In contrast, a one log_10_ drop of HPgV-1 titers at week 2 was observed in 10 patients during treatment with sofosbuvir-containing regimens, sustained to the end of treatment (EOT) and in two cases decreasing to below the detection limit of the assay. For the five patients treated with ledipasvir/sofosbuvir with the inclusion of pegylated interferon, titers decreased to below the detection limit at week 2 and remained undetectable to EOT. Subsequently, the HPgV-1 titer rebounded to pretreatment levels for all patients. In conclusion, we found that HCV treatment regimens that included the polymerase inhibitor sofosbuvir resulted in decreases in HPgV-1 titers, and the addition of pegylated interferon increased the effect on patients with coinfections. This points to the high specificity of protease and NS5A inhibitors toward HCV and the more broad-spectrum activity of sofosbuvir and especially pegylated interferon.

**IMPORTANCE:**

Human pegivirus 1 coinfections are common in hepatitis C virus (HCV) patients, persisting for years. However, little is known about how pegivirus coinfections are affected by treatment with pangenotypic direct-acting antivirals (DAAs) against HCV. We identified human pegivirus by metagenomic analysis of chronic HCV patients undergoing protease, NS5A, and polymerase inhibitor treatment, in some patients with the addition of pegylated interferon, and followed viral kinetics of both viruses to investigate treatment effects. Only during HCV DAA treatment regimens that included the more broad-spectrum drug sofosbuvir could we detect a consistent decline in pegivirus titers that, however, rebounded to pretreatment levels after treatment cessation. The addition of pegylated interferon gave the highest effect with pegivirus titers decreasing to below the assay detection limit, but without clearance. These results reveal the limited effect of frontline HCV drugs on the closest related human virus, but sofosbuvir appeared to have the potential to be repurposed for other viral diseases.

## INTRODUCTION

Human pegivirus 1 (HPgV-1) is a single-stranded positive-sense RNA virus of the *Pegivirus* genus within the *Flaviviridae* family (1, 2). It was formerly known as GB virus C or hepatitis G virus and is the closest related human virus to hepatitis C virus (HCV). HCV causes chronic liver infection, and long-term persistent infection can result in liver cirrhosis and hepatocellular carcinoma leading to up to 300,000 deaths annually, worldwide (3, 4). HCV is a positive-sense RNA virus with one open reading frame (ORF) coding for a polyprotein that is subsequently cleaved into 10 mature proteins (5); HPgV-1 is believed to have a similar genome organization. HPgV-1 infections in humans are commonly seen with prevalence in healthy blood donors of 1%–4% (1, 6). Usually, HPgV-1 infections last for more than 6 months, but more than 50% of infected individuals will experience viral clearance within 2 years (7–9). Infection with this prevalent virus has not been definitively associated with any acute or chronic human diseases (10, 11). Nor did it lead to hepatitis or any other symptoms in experimentally infected chimpanzees (12). However, it has been reported to increase the risk for non-Hodgkins lymphoma (13–16). Further, it has been suggested to be associated with encephalitis in a few patients (17). HPgV-1 and HCV share the same transmission routes, and coinfection is, therefore, frequently seen with a reported prevalence between 10% and 25% (18, 19). Recently, the advances in sequencing technology and the use of metagenomic unbiased approaches have allowed the identification of diverse viral infections (20), and in addition, this approach can detect and sequence HPgV-1 (19). No correlation between coinfection and HCV disease progression or effect on the outcome of HCV treatment with antivirals has been found (21).

The development of direct-acting antivirals (DAAs) for HCV has led to a revolution in treatment with cure rates above 90% (22, 23). The treatment regimens always contain an NS5A inhibitor [pibrentasvir (PIB), velpatasvir (VEL), or ledipasvir (LED)] and either a NS3/4A protease inhibitor glecaprevir (GLE) (name used in clinic Maviret) or the nucleotide analog sofosbuvir (SOF) inhibiting NS5B (name used in clinic Epclusa or Harvoni). A triple combination is also available containing all three classes of drugs [voxilaprevir (VOX), velpatasvir, and sofosbuvir; name used in clinic Vosevi] (24). While the NS5A inhibitors are greatly diminished in potency for the related rat hepacivirus (RHV) and NS3/4A protease inhibitors had no effect, sofosbuvir could suppress RHV replication (25). In addition, it was reported that NS3/4A protease inhibitors had no effect on the protease activity of HPgV-1 (26). Since HPgV-1 has homologs of HCV mature proteins NS3/4A, NS5A, and NS5B, the virus could potentially be affected by HCV treatment or immune system activation during HCV clearance. In the era before the introduction of DAAs, interferon was used alone or in combination with ribavirin (RBV) to treat HCV. In that connection, coinfection studies with HPgV-1 found that interferon had the potential to clear the pegivirus coinfection (27–30).

In this study, we investigated coinfections of HPgV-1 in chronic HCV-infected patients, identified and analyzed by a metagenomic approach or reverse transcriptase-quantitative PCR (RT-qPCR), and quantitively measured longitudinally by RT-qPCR. In these patients, it was possible to investigate the effect of several different DAA regimens on plasma HPgV-1 viral titers due to longitudinal sampling before, under, and after treatment. In addition, we could compare regimens with and without sofosbuvir, ribavirin, or pegylated interferon (PEG-INF), considered more broad-spectrum antivirals, and their effect on HPgV-1.

## MATERIALS AND METHODS

### Chronic hepatitis C patients and treatment history with DAA

A subset of the patients included in this study (group 1) were originally from a randomized trial to investigate the sustained virological response at week 12 (SVR12) following 4 weeks of treatment with GLE/PIB ± RBV; treatments did not include PEG-INF (31). In this article, the patient IDs for these patients are marked with an A as a prefix in the text and figures. All included patients were treatment-naïve and had an absence of liver fibrosis defined as liver stiffness measurement by transient elastography <8 kPa. No patients had any coinfection with hepatitis B virus or HIV, and 75% had a history of intravenous drug use. All patients with virological relapse were characterized as treatment failures and retreated with 12 weeks of sofosbuvir-containing regimens. Similarly, patients were included from a prior trial (group 2) to disseminate SVR12 following 4 weeks of treatment with LED/SOF with RBV and ± PEG-INF (32). This study was conducted at an outreach drug treatment center where included participants were individuals with a history of intravenous drug use with the same inclusion criteria as group 1, but HCV baseline viral load should be below 2,000,000 IU/mL. The IDs of these patients are marked with a W as a prefix in text and figures. From a third cohort (group 3), which includes patients with chronic hepatitis C who are treated with DAAs at the Department of Infectious Diseases, Copenhagen University Hospital, Hvidovre, patients were selected who had plasma samples available at the baseline, week 2, the end of treatment (EOT), and 12 weeks after EOT. For 28 patients, HCV RNA sequence data were available from previous HCV genotyping at the Department of Microbiology, Copenhagen University Hospital, Hvidovre. Pegivirus-positive patients from this group are marked with a T as a prefix in the text and figures. Two patients had liver cirrhosis and were treated with SOF/VEL for 12 weeks, while one patient had mild fibrosis with a transient elastography of 5.4 kPa and was treated with GLE/PIB/SOF for 12 weeks. None of the patients were coinfected with HIV or hepatitis B virus, and all patients achieved SVR12.

### HCV and HPgV-1 RT-qPCR

For patient groups 1 and 2, HCV RNA viral load was determined from plasma samples using the Cobas HCV assay, run on the 6800/8800 systems (Roche Molecular System, Inc.), as described previously (31, 32). All analyses were performed according to the manufacturer’s instructions. The lower detection limit of the Cobas HCV assay was 15 IU/mL. HCV RNA in plasma samples from the patients in group 3 was quantified using the Aptima HCV Quant Dx Assay (Hologic Inc, San Diego, CA, USA) with a lower limit of quantification at 10 IU/mL, as previously described (33). For HPgV-1, RNA was extracted as described earlier (34). The RT-qPCR procedure for HPgV-1 was adapted from reference (35) to TaqMan Fast Virus 1-Step Master Mix (ThermoFisher, Waltham, MA, USA). The assay was run on a Lightcycler 96 (Roche, Basel, Switzerland) and analyzed with the Lightcycler 96 software (Roche, Basel, Switzerland) version 1.10.1320. HPgV-1 genome equivalents per milliliter (GE/mL) were calculated by interpolation for an in-run standard curve. The in-run standard was patient RNA run in a 10-fold dilution series with the absolute concentration extrapolated from RNA-seq sequence coverage of both HCV and HPgV-1 from group 1 and HCV RNA titers measured by RT-qPCR determined above.

### Viral genome sequencing and data analysis

As reported earlier, RNA was extracted from the patient plasma samples (31, 34) and sequenced on an Illumina NextSeq or Miseq platform (31). Sequencing was performed for all patients at the baseline and the time of virological relapse in those with HCV treatment failure. Data were analyzed by initial human sequence depletion and subsequent *de novo* assembly identifying both HCV and HPgV-1 genomes, and subsequently, reads were mapped on the assembled genome to further refine the sequence and investigate the viral population (36–39). ORF sequences of the pegiviruses were aligned with relevant reference sequences using MAFFT, and phylogeny was built by PhyML applying the general time reversible substitution model. One hundred bootstraps were performed to consolidate the phylogeny. Population selection analysis was performed using the SNPGenie tool with a sliding window range of 15 amino acids, in order to calculate pairwise distances.

## RESULTS

### Metagenomic analysis of RNA-seq data from HCV patients revealed frequent HPgV-1 coinfections

Samples from 32 chronic hepatitis C patients (group 1) treated with 4 weeks of GLE/PIB ± RBV (31), 28 patients (group 2) treated with 4 weeks of LED/SOF + RBV ± PEG-INF (32), and 28 patients (group 3) with various DAA treatments were analyzed by metagenomics and RT-qPCR. The vast majority of these 88 patients had samples available for analysis before, during, and after treatment for chronic hepatitis C.

Sequencing data from the 32 samples from patients in group 1 underwent human genomic sequence depletion and subsequent *de novo* assembly to create contigs. Besides the HCV contigs described previously (31), we found that 10 (31%) had non-HCV contigs above 9 kb with sufficient coverage (Table 1).

**TABLE 1:** Patient HPgV-1 sequencing contig and coverage parameters

Patient ID	HCV treatment outcome	Genotype	Length of contig	Coverage of genome (%)	Average depth	GenBank accession no.
A102	SVR	2	9,367	99.7	8,138	PP783765
A103	SVR	2	9,380	99.9	144,268	PP783766
A106	Failure	1	9,386	99.9	171,647	PP783767
A112	SVR	2	9,395	99.9	268,890	PP783768
A117	Failure	2	9,383	99.9	245,100	PP783769
A119	Failure	1	9,386	99.9	66,103	PP783770
A123	SVR	2	9,391	99.9	186,664	PP783771
A124	Failure	2	9,383	99.9	140,452	PP783772
A125	Failure	2	9,386	99.9	174,703	PP783773
A126	SVR	2	9,387	99.9	357,073	PP783774
W101	SVR	2	9,204	98.0	617	PP783778
W108	SVR	2	9,228	98.2	5,820	PP783779
W130	SVR	2	8,664	92.7	2,410	PP783780
W133	SVR	2	8,929	95.1	5,576	PP783781
W136	Failure	2	9,384	99.9	45,367	PP783782
W143	SVR	2	9,371	99.8	12,361	PP783783
W145	SVR	2	9,378	99.8	33,304	PP783784
T134	SVR	2	9,345	99.5	13,057	PP783775
T167	SVR	2	9,376	99.8	12,998	PP783776
T213	SVR	2	9,362	99.7	27,432	PP783777

Among the 28 group 2 patients, 8 (28%) were found positive for HPgV-1 by RT-qPCR, and the 7 patients with samples available throughout treatment were similarly sequenced as the 10 group 1 patients described above (Table 1).

Finally, 28 patients in group 3 were HCV-genotyped by RNAseq and screened for HPgV-1, and 3 (11%) patients were found positive for HPgV-1 (Table 1).

**Fig 1:**
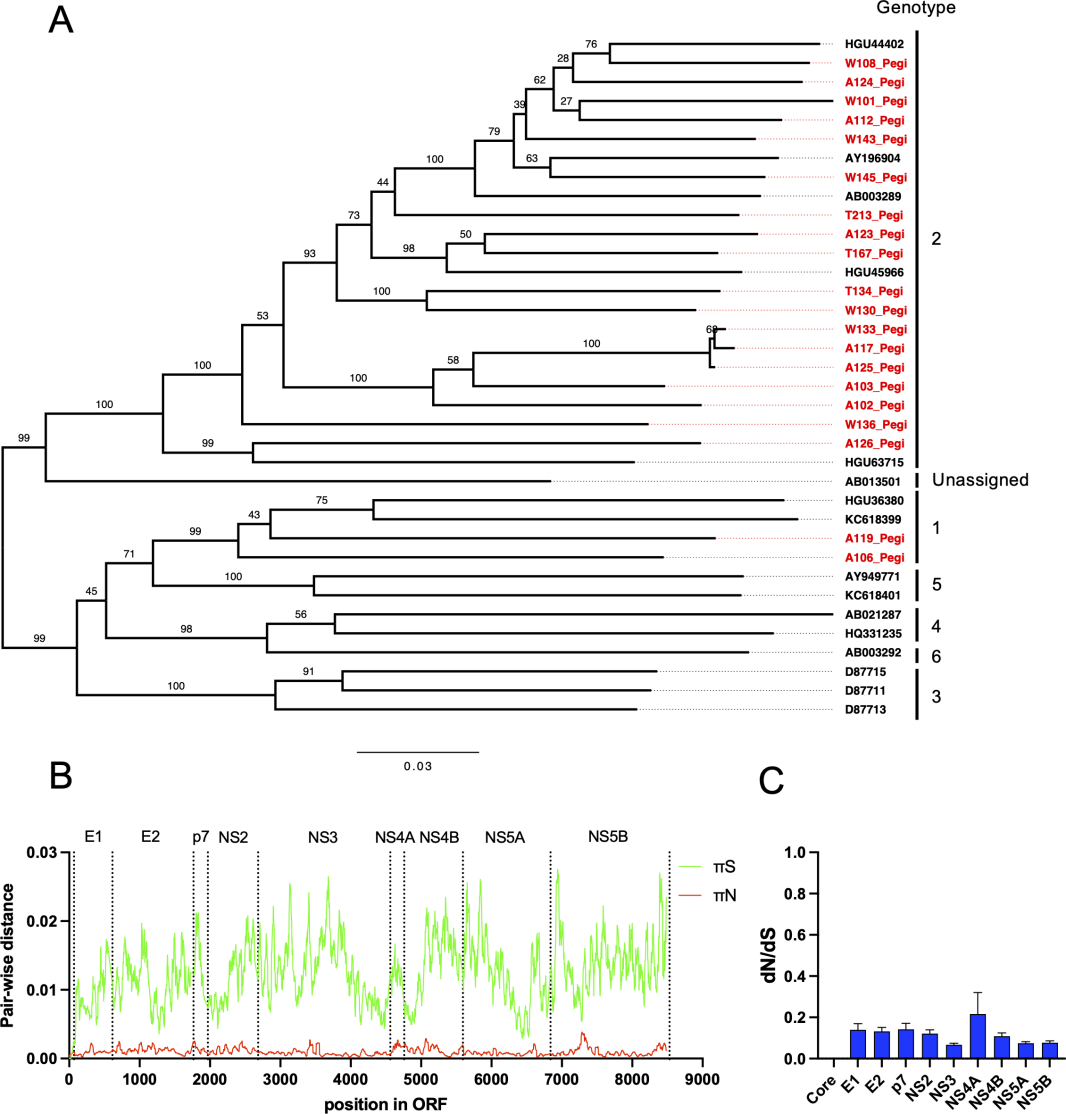
Phylogenetic and selection analysis of the human pegivirus genome population. (**A**) Phylogenetic tree of full-length human pegivirus ORF sequences recovered from 20 HCV patients included in this study and relevant pegivirus reference sequences of genotypes 1–6. The tree is midpoint-rooted, and pegivirus genotypes are indicated at the tips. The scale bar depicts changes per site, and branch labels represent bootstrap support (*n* = 100). The prefix letters A, W, and T correspond to the three patient study groups defined in Materials and Methods. (**B**) New synonymous and non-synonymous π (pairwise distance) across the complete pegivirus ORF; the data are averaged for HPgV-1 recovered from the 18 out of 20 included HPgV-1/HCV coinfected patients (W101 and W130 were not included since they did not have sufficient coverage to be included, below 5,000 on average as seen in Table 1) in a sliding window of 15 amino acids. A junction of each predicted mature protein is indicated as vertical dotted lines with the protein label shown above. The small space between the translation start and E1 is considered the core region. (**C**) dN/dS ratios of each predicted mature pegivirus protein shown as averages of the HPgV-1 recovered from 18 out of 20 HPgV-1/HCV coinfected HCV patients (W101 and W130 were not included since they did not have sufficient coverage as stated above) with SEM depicted as error bars.

When taking all the above patients into consideration, the HPgV-1 coinfection status was not known when the HCV treatment was initiated but discovered in the subsequent sequence analysis and by RT-qPCR. All non-HCV sequences were identified as HPgV-1 (Fig. 1A), and almost the entire genome was covered, except for the last bases at the 3′ end, and for all 20 HPgV-1 samples, a mean sequencing depth of coverage from 5,000 to over 100,000 was observed except for two samples that fell below 5,000; these two sequences were not analyzed in the data shown in Fig. 1B and C (Table 1).

When linking the treatment outcome in the 32 patients treated for 4 weeks with GLE/PIB ± RBV, we found 5 coinfections out of 11 (46%) experiencing HCV treatment failure and 5 coinfections out of 21 (24%) with SVR, but this difference was not significant.

Phylogenetic analysis at baseline revealed that the identified human pegiviruses belong to genotypes 1 and 2, and we observed a monophyletic group within genotype 2 only distantly related to the rest of the genotype 2 sequences, which could form a regional cluster found in Denmark (Fig. 1A). Patients A117, A125, and W133 were found to have sequences of such close relatedness that it could indicate a common transmission cluster. Selection analysis at baseline within the virus populations revealed the HPgV-1 genome to be under negative selective pressure with no obvious hotspots for adaptation (Fig. 1B). Individual mature proteins all showed similarly low dN/dS ratios with no distinction between them, except NS4A, that seems to be under less strict negative selection although not significant (Fig. 1C).

### HCV DAA treatment regimens containing the nucleotide analog sofosbuvir and especially pegylated interferon had the greatest influence on HPgV-1 titers

**Fig 2:**
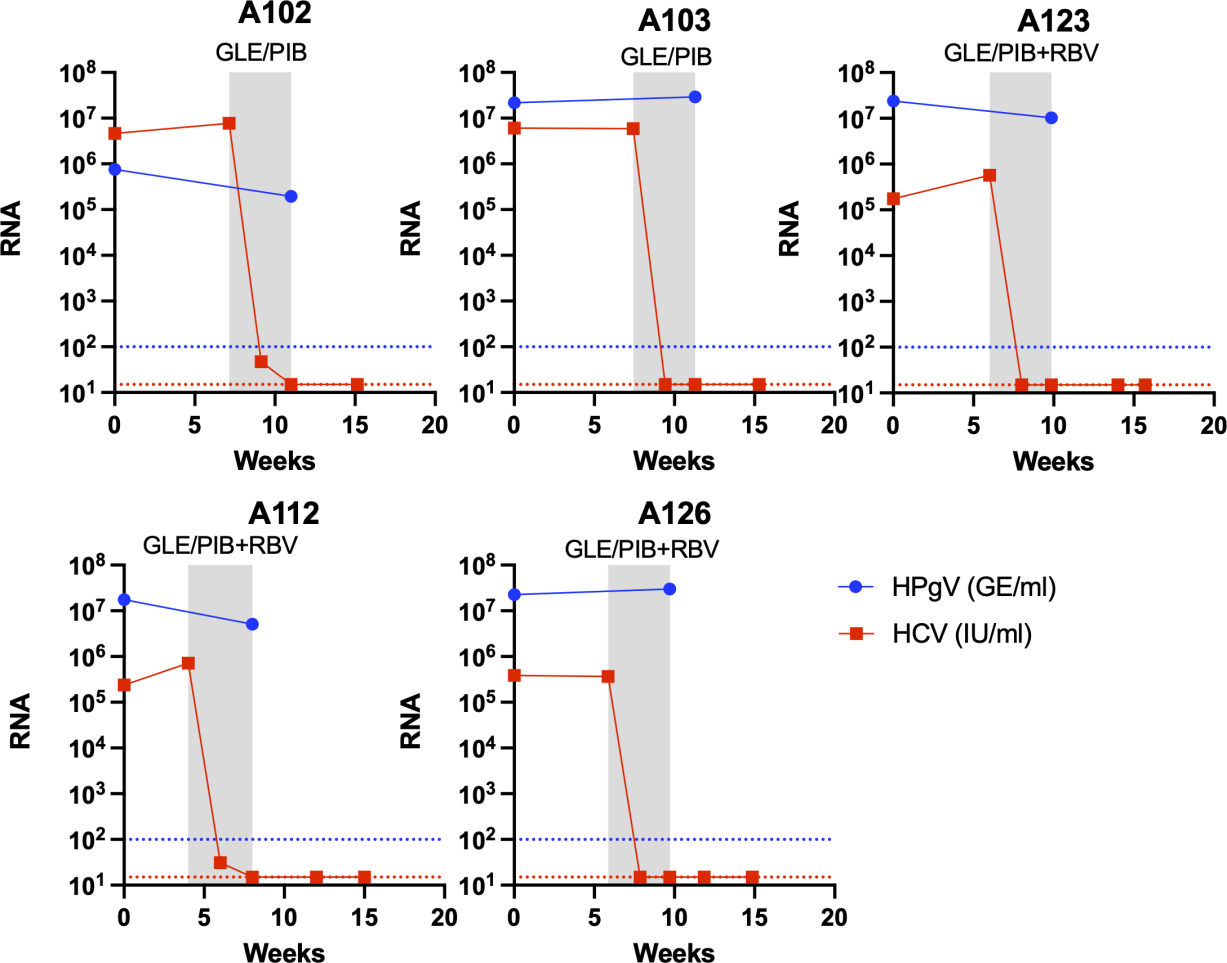
Viremia development of human pegivirus during glecaprevir/pibrentasvir treatment in HCV patients with SVR. RNA titer measurements from HCV patients treated with GLE/PIB of HCV and HPgV-1 in plasma are shown at the *y*-axis as international units per milliliter (IU/mL) or genome equivalents per milliliter (GE/mL), respectively, and time in weeks at the *x*-axis. The gray boxes indicate periods during antiviral treatment with the type of treatment indicated at the top. Ribavirin addition to the treatment is indicated as RBV. Each panel represents an SVR patient. A red titer line at the *y*-axis represents undetectable HCV RNA titers (limit of detection 15 IU/mL). A blue titer line at the *y*-axis represents undetectable HPgV-1 RNA titers (limit of detection 100 GE/mL).

**Fig 3:**
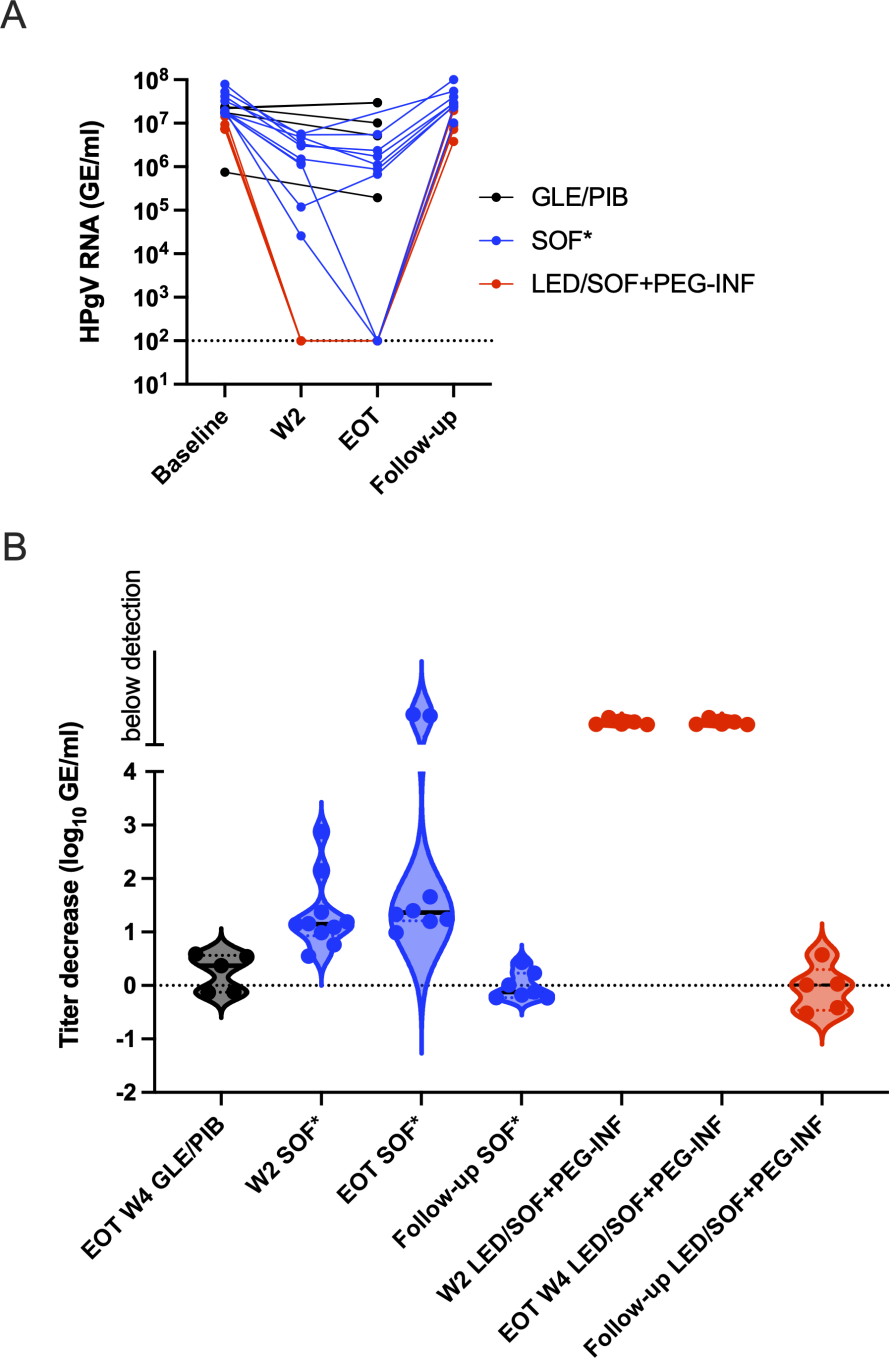
Human pegivirus viremia development during treatment of HCV with DAA without or with sofosbuvir. (**A**) Individual patient HPgV-1 RNA titer in plasma shown as connected dots with timepoint indicated on the *x*-axis in SVR HCV patients during GLE/PIB treatment, HCV patients during sofosbuvir-containing treatment referred to as SOF*, including LED/SOF, VEL/SOF, VOX/VEL/SOF, GLE/PIB/SOF, and HCV patients during LED/SOF plus PEG-INF. (**B**) HPgV-1 RNA titer changes in treated patients compared to their respective baseline samples shown as log_10_ GE/mL drop on the *y*-axis and depicted as violin plots. Groupings are identical to (A).

**Fig 4:**
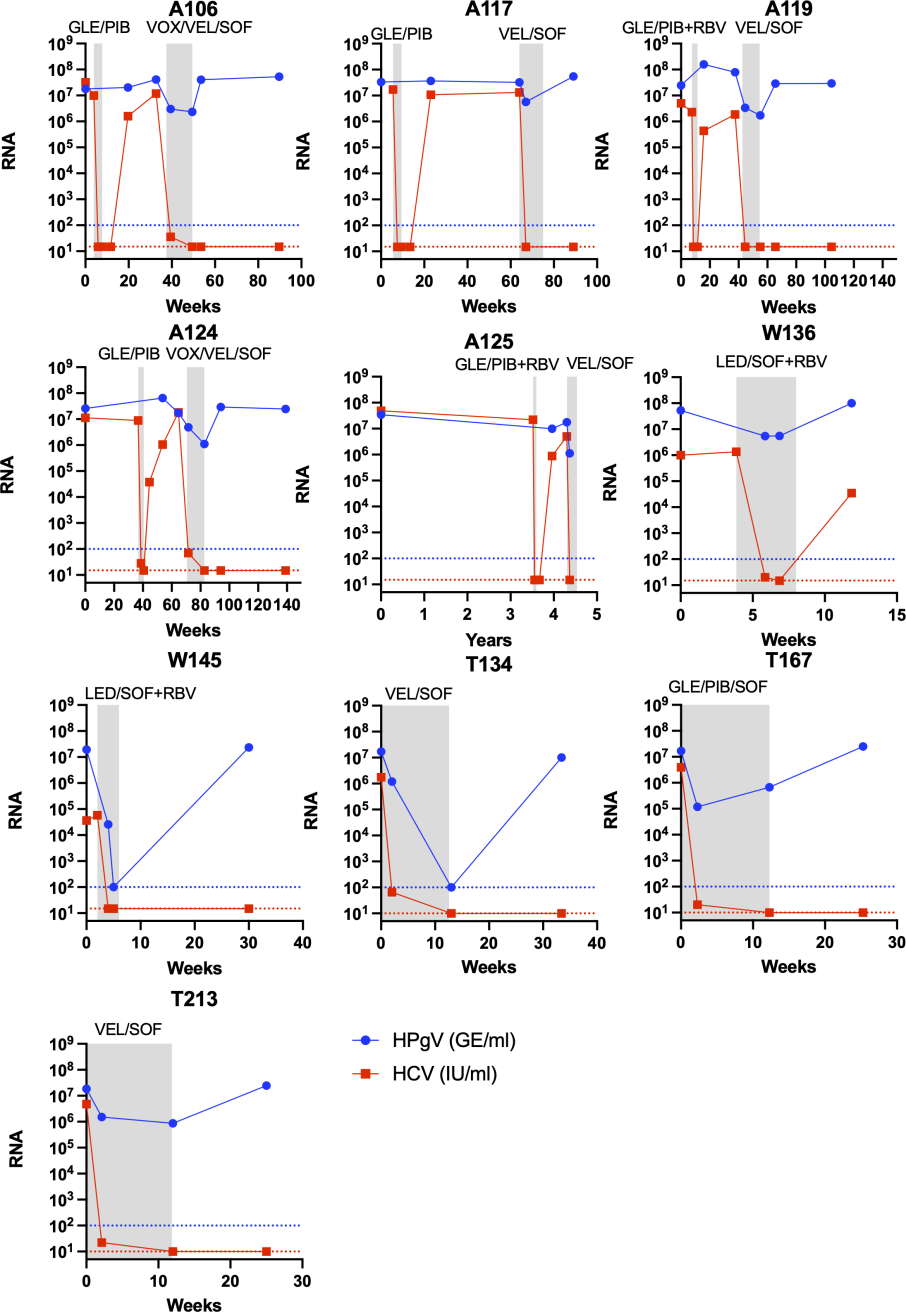
RNA titer decrease of HCV and HPgV-1 in HCV patients during treatment with sofosbuvir-containing regimens. RNA titer measurements in plasma of HCV and HPgV-1 are shown at the *y*-axis as international units per milliliter (IU/mL) or genome equivalents per milliliter (GE/mL), respectively, and time at the *x*-axis. The gray boxes indicate periods during antiviral treatment with the type of treatment indicated at the top. Each panel represents a patient treated with regimens containing SOF, including LED/SOF, VEL/SOF, VOX/VEL/SOF, and GLE/PIB/SOF. A red titer line representing HCV RNA at the *y*-axis represents undetectable HCV RNA titers (limit of detection 15 IU/mL except for patients T134, T167, and T213 with limit of detection 10 IU/mL). Ribavirin addition to the treatment is indicated as RBV. A blue titer line representing HPgV-1 RNA at the *y*-axis represents undetectable HPgV-1 RNA titers (limit of detection 100 GE/mL).

**Fig 5:**
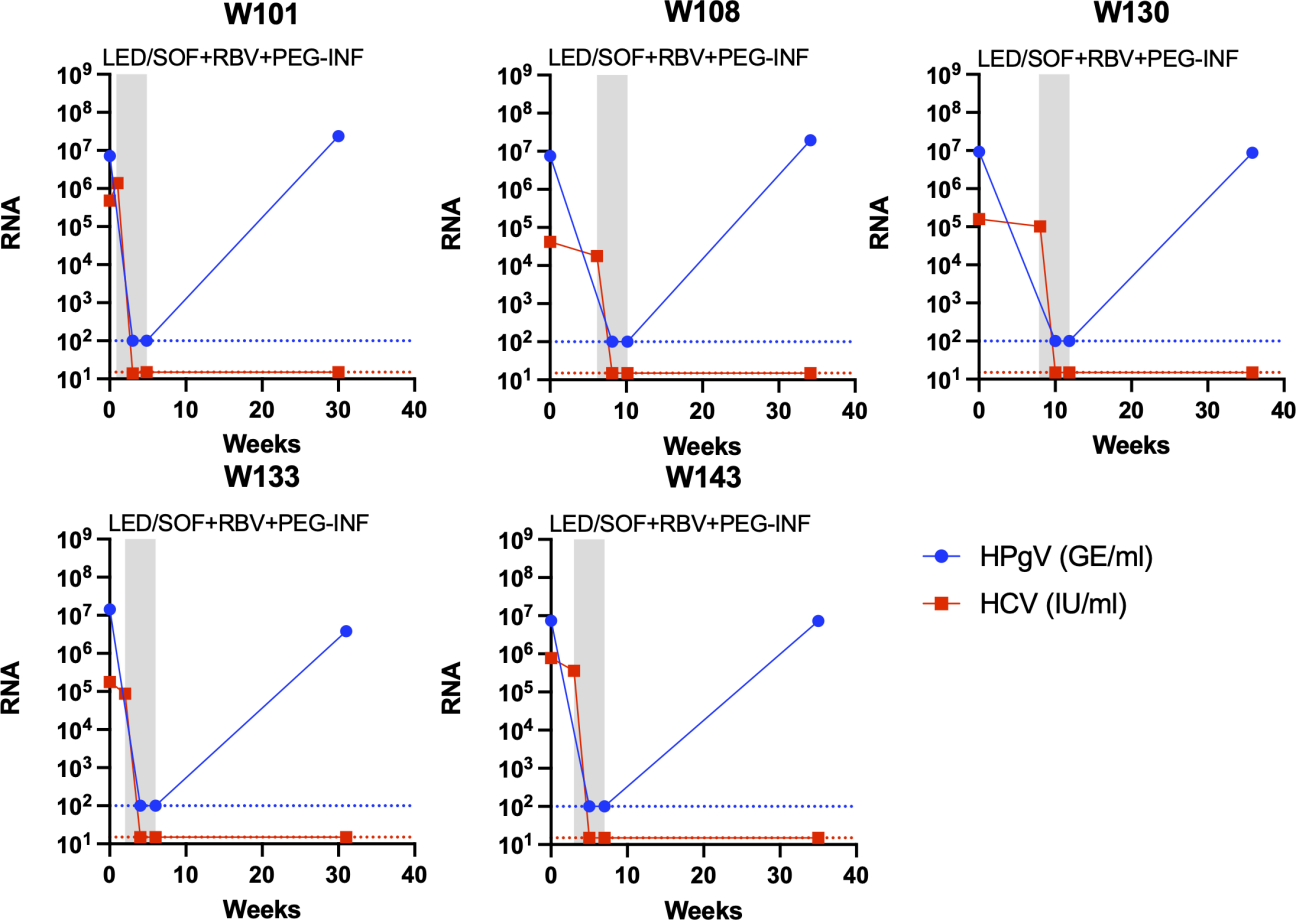
Viremia development of HCV and HPgV-1 in HCV patients during treatment with a sofosbuvir and pegylated interferon-containing regimen. RNA titer measurements in plasma of HCV and HPgV-1 are shown at the *y*-axis as international units per milliliter (IU/mL) or genome equivalents per milliliter (GE/mL), respectively, and time at the *x*-axis. The gray boxes indicate periods during antiviral treatment with the type of treatment indicated at the top. Each panel represents a patient treated with LED/SOF plus RBV and PEG-INF. A red titer line representing HCV RNA at the *y*-axis represents undetectable HCV RNA titers (limit of detection 15 IU/mL). A blue titer line representing HPgV-1 RNA at the *y*-axis represents undetectable HPgV-1 RNA titers (limit of detection 100 GE/mL).

All 20 HPgV-1 coinfections were confirmed by RT-qPCR with titers of between 10^7^ and 10^8^ GE/mL, except one sample that was below 10^6^ GE/mL. For the five HCV SVR patients, HCV RNA titers became undetectable at the end of the 4 weeks of GLE/PIB ± RBV treatment. However, no clear pattern of decrease in titer was observed for HPgV-1 RNA at the EOT, with three patients having a fivefold drop while two patients had a small titer increase, even with the addition of RBV to the treatment (Fig. 2, 3A and B). For the 10 patients treated with SOF-containing regimens, longitudinal samples were available for analysis by RT-qPCR before, during (week 2), at EOT, and at follow-up of the SOF-containing treatment. HCV RNA in the plasma became undetectable between week 2 and EOT for all patients. Further, for all 10 patients, a 10-fold drop in the HPgV-1 RNA titers compared to baseline could be observed at week 2, and the titers decreased further to about 20-fold at EOT and in two cases to below the detection level (Fig. 3A and B, Fig. 4). During follow-up, HPgV-1 RNA rebounded to titers as observed before treatment for the nine patients with samples available (Fig. 3A and B, Fig. 4). In contrast, the five patients treated with LED/SOF+ RBV + PEG-INF had a dramatic drop in HPgV-1 titers that fell to below the detection limit at week 2 during treatment and stayed below detection at EOT at week 4 (Fig. 3A and B, Fig. 5). However, the titers rebounded in the follow-up to pretreatment levels. Thus, permanent clearance of HPgV-1 was not detected in any of the treated patients.

## DISCUSSION

In this study, we identified HCV and HPgV-1 coinfections in patients with chronic HCV by metagenomic analysis and confirmed findings by RT-qPCR targeting the individual viruses. A subset of patients had received 4 weeks of the GLE/PIB ± RBV (Maviret) protease/NS5A inhibitor combination, and patients with treatment failure and viral relapse were retreated with polymerase inhibitor SOF-containing regimens either with NS5A inhibitor velpatasvir (in Epclusa) or velpatasvir and protease inhibitor voxilaprevir (in Vosevi) (24). In addition, patients treated with 4 weeks of NS5A inhibitor LED and polymerase inhibitor SOF (in Harvoni) + RBV ± PEG-INF were analyzed (32). Finally, HCV patients coinfected with HPgV-1, identified from the clinic, and treated with SOF-containing regimens were included. This allowed the assessment of the antiviral effect of these different treatments on the coinfecting HPgV-1 virus.

Metagenomic analysis of viral coinfections is a powerful tool, and having sufficient read depth not only permitted robust detection, as shown by others (19, 40), but also allowed us to address viral population composition and evolution. We found a high prevalence of coinfections with HPgV-1 in patient groups 1 and 2, with frequencies of 31% and 28%, respectively, compared to other reports where they found 10%–25% (18, 19). This probably reflects the fact that the majority of these patients have high-risk behavior, such as a history of intravenous drug use. In contrast, we only identified 11% coinfections when screening patients from group 3, which could reflect the overall prevalence in the HCV-infected patients from Denmark. While there clearly was a cluster of HPgV-1 sequences within genotype 2 that could form a Danish clade, only three patients had sequences of such close relation that this could indicate a transmission event. The rest were scattered within genotypes 1 and 2 that reflect the worldwide HPgV-1 genotype distribution with genotype 2 being the most prevalent in Europe (41).

We found 24% and 46% HPgV-1 coinfections in SVR and failure patients, respectively, treated with 4 weeks of GLE/PIB ± RBV. Although there was no significant difference in the coinfection frequency observed between the groups, there is a possibility that coinfections could affect 4 weeks of GLE/PIB treatment. However, out of the 28 patients treated with LED/SOF + RBV ± PEG-INF, only one coinfected patient (W136) experienced treatment failure, thus further not supporting any connection between outcome and coinfection status. This is in accordance with a previous study (21). However, no screening was performed before treatment initiation, and therefore, we cannot rule out that HCV patients with HPgV-1 coinfections might be less susceptible to GLE/PIB treatment. This contrasts coinfections with HIV, where a correlation between less severe disease outcomes and HPgV-1 infection was reported (42). Larger patient data sets are needed to further evaluate the influence of HPgV-1 coinfection on HCV treatment outcomes. However, it is not easy to explain the mechanism of how coinfections could affect the treatment outcome due to the different compartments of replication of HCV and HPgV-1.

Overall, the HPgV-1 genomes of different patients were under negative selection with most of the intra-population diversity being synonymous mutations, and no hotspots of non-synonymous changes could be observed. This is in concordance with previous analysis of viral evolution also showing similar dN/dS ratios (19) and points toward the HPgV-1 virus presence being undetected by the immune system and, therefore, not developing escape mutations. This was further supported by sequence analysis from horses experimentally infected with the equine pegivirus (43). Particularly, the proposed small core region had almost no diversity, indicating a vital function in viral assembly and high sequence conservation important for assembly, as previously proposed (44).

While the 4 weeks of GLE/PIB ± RBV treatment cured 66% (21/32) of the HCV patients (31), none of the 10 coinfected cleared their HPgV-1 infection. In addition, of the five patients with HCV SVR, HPgV-1 viremia titer decreased to 1/5 after 4 weeks (at EOT) for three patients while HPgV-1 viremia titer did not decrease for two patients. This pointed toward the limited efficacy of protease inhibitor GLE and NS5A inhibitor PIB toward HPgV-1. The addition of RBV did not show any effect on the HPgV-1 titers and has shown only a slight effect on HCV titers in monotherapy (45). In contrast, treatment with SOF-containing regimens in ten HCV patients led to a 10-fold decrease in HPgV-1 viremia titer when compared to baseline at week 2 and a further small reduction to 20-fold at EOT or in two cases below the detection limit of the titer assay. Titers rebounded to pretreatment values after treatment was ended. Although these regimens also contained the NS5A inhibitors VEL and LED and in some cases the NS3 protease inhibitor VOX, these inhibitors are very similar to PIB and GLE in their mode of action, respectively, and, therefore, not likely to be the inhibiting factors. In addition, patient T167 treated with PIB/GLE and SOF had an almost 100-fold reduction at week 2 supporting SOF to be the effective agent. Taken together, the broad-spectrum nucleotide analog SOF appeared to exhibit the highest antiviral effect against HPgV-1, while the more HCV-specific protease and NS5A inhibitors had minimal antiviral effect on this virus. The activity of HCV protease inhibitors in severe acute respiratory syndrome coronavirus 2 has been shown *in vitro* (46). Although SOF, thus, seems to have some antiviral effect against HPgV-1, the potency of the drug is diminished indicating structural and functional differences in NS5B compared to HCV. This is similar to what has been reported for the HCV-related RHV virus, where only SOF had an effect on replication and could decrease viremia titers in rats (25, 47). A study reported similar effects of the SOF-containing regimens as we observed at EOT in coinfected HCV patients, with the only sustained suppression observed in two other patients treated with telaprevir/SOF and interferon (48). A case study of an HCV patient reported a reduction in HPgV-1 viral RNA during SOF-containing DAA treatment (40), and a similar drop during SOF treatment was reported for a few cases of HPgV-2 HCV coinfections (49). Sofosbuvir has been used in clinical studies to treat chronically infected hepatitis E patients; a pilot monotherapy clinical trial study showed only a 1 log drop in RNA titer and later viral rebound during treatment (50). No clearance was observed, but some effect was seen in the clinical parameters such as a drop in the alanine transaminase level, and resistance-associated variants seemed to arise in some of the patients (51). These results have also been supported by several single case studies (52–54). Sofosbuvir has also shown potential against tickborne encephalitis virus and yellow fever virus in cell culture with low IC_50_ ratios (55) and for yellow fever virus through *in vivo* mouse experiments (56). In addition, the drug has shown to positively affect disease outcomes in clinical yellow fever virus patients (57). The addition of PEG-INF had a clear effect on the LED/SOF treatment regimen with a drop in viremia below detection at week 2 and sustained until EOT, and interferon was reported to have the potential to clear pegivirus coinfections (27–30). However, 4 weeks seems to be too short a treatment period since we did not observe permanent clearance in any of the patients in our study. The lack of robust *in vitro* cell culture systems for HPgV challenges the systematic testing of the individual drugs.

The main limitation of the present study is the limited number of patients, as also mentioned above. However, these are unique patient samples that are matched and analyzed before, during, and after treatment. In addition, this was a special opportunity to explore HPgV-1, the closest related human virus to HCV, response to DAA treatment in patients and how HPgV-1 coinfection affects HCV treatment.

In summary, we found that 20 out of 88 (23%) screened HCV patients in this study had a HPgV-1 coinfection, belonging to genotypes 1 and 2. Sofosbuvir-containing regimens decreased the HPgV-1 viral titer in the blood. Further, our data confirmed a profound effect of PEG-INF on HPgV-1 viral titers. Whether HPgV-1 coinfection affects HCV disease progression or clearance remains elusive, and more research into this seemingly harmless HPgV-1 chronic infection is needed to determine if it is indeed harmless and what role it plays in coinfection. The limited effect observed of frontline HCV drugs, except for SOF, on the closest related human virus suggests primarily the potential of repurposing SOF against other viral infections.

## Data Availability

The raw data sets presented in this article are not readily available because they contain identifiable human genome sequences. Requests to access the data sets should be directed to the corresponding authors.
